# Endothelin Receptor A Blockade Is an Ineffective Treatment for Adriamycin Nephropathy

**DOI:** 10.1371/journal.pone.0079963

**Published:** 2013-11-12

**Authors:** Roderick J. Tan, Lili Zhou, Dong Zhou, Lin Lin, Youhua Liu

**Affiliations:** 1 Department of Medicine, University of Pittsburgh School of Medicine, Pittsburgh, Pennsylvania, United States of America; 2 Department of Pathology, University of Pittsburgh School of Medicine, Pittsburgh, Pennsylvania, United States of America; Institut National de la Santé et de la Recherche Médicale, France

## Abstract

Endothelin is a vasoconstricting peptide that plays a key role in vascular homeostasis, exerting its biologic effects via two receptors, the endothelin receptor A (ETA) and endothelin receptor B (ETB). Activation of ETA and ETB has opposing actions, in which hyperactive ETA is generally vasoconstrictive and pathologic. Selective ETA blockade has been shown to be beneficial in renal injuries such as diabetic nephropathy and can improve proteinuria. Atrasentan is a selective pharmacologic ETA blocker that preferentially inhibits ETA activation. In this study, we evaluated the efficacy of ETA blockade by atrasentan in ameliorating proteinuria and kidney injury in murine adriamycin nephropathy, a model of human focal segmental glomerulosclerosis. We found that ETA expression was unaltered during the course of adriamycin nephropathy. Whether initiated prior to injury in a prevention protocol (5 mg/kg/day, i.p.) or after injury onset in a therapeutic protocol (7 mg/kg or 20 mg/kg three times a week, i.p.), atrasentan did not significantly affect the initiation and progression of adriamycin-induced albuminuria (as measured by urinary albumin-to-creatinine ratios). Indices of glomerular damage were also not improved in atrasentan-treated groups, in either the prevention or therapeutic protocols. Atrasentan also failed to improve kidney function as determined by serum creatinine, histologic damage, and mRNA expression of numerous fibrosis-related genes such as collagen-I and TGF-β1. Therefore, we conclude that selective blockade of ETA by atrasentan has no effect on preventing or ameliorating proteinuria and kidney injury in adriamycin nephropathy.

## Introduction

Upregulation of endothelin signaling has been implicated in a wide variety of chronic kidney diseases (CKD). Endothelins are 21-amino acid peptides that act as potent vasoconstrictors, and there are three known isotypes (ET-1, ET-2, and ET-3). The kidney is a major source of ET-1, with most cells in the kidney capable of producing the pre-pro-endothelin-1 peptide that is subsequently processed to the final 21-amino acid form [1]. Current evidence suggests important roles for ET-1 in regulating proteinuria, systemic blood pressure, intraglomerular pressures, and CKD progression [2].

Endothelins exert biologic effects via two receptors, endothelin receptor A and endothelin receptor B (ETA and ETB). Since ETA activation leads to vasoconstriction, it is widely thought that ETA contributes to renal pathology. Meanwhile, ETB activation may be protective, leading to nitric oxide release and vasorelaxation [3]. As such, specific ETA receptor inhibitors have been developed in an attempt to block the pathologic activation of this receptor while sparing ETB receptor signaling. 

Atrasentan is a selective endothelin receptor inhibitor with a 1000 to 2000-fold greater affinity for ETA compared to ETB. This selectivity has been exploited in experimental models, in which atrasentan has been shown to inhibit various kidney injuries [4]. For instance, atrasentan was capable of inhibiting proteinuria, inflammation, and glomerular permeability, while abrogating declining nephrin expression in experimental diabetes in rodents [5]. Atrasentan could also ameliorate hypertensive kidney disease with a magnitude comparable to renin-angiotensin system (RAS) blockade [6]. Other specifically-designed ETA inhibitors such as avosentan and sitaxsentan were similarly protective in diabetic nephropathy and 5/6 nephrectomy models [7-9]. 

Human trials have shown promise in treating kidney disease as well. When added to the standard of care (RAS inhibitors) in diabetic patients, ETA blockade with either atrasentan or avosentan was capable of reducing proteinuria, a key indicator of renal injury [10-12]. However, adverse side effects, in particular fluid retention, have limited the use of these agents in the clinic. 

In this study we attempted to further examine the role of atrasentan in proteinuria and podocyte injury. To do this, we utilized the adriamycin nephropathy model of proteinuria and glomerular injury, which recapitulates the human disease of focal segmental glomerulosclerosis (FSGS). In this model, adriamycin administration causes glomerular damage and increased urinary albumin excretion, followed by tubular atrophy and dilation and ultimately renal fibrosis in mice [13]. We hypothesized that atrasentan can inhibit the development of proteinuria in this model similar to the diabetic mouse model and human trials. However, we found that atrasentan given before the onset or after the initiation of kidney injury could not prevent adriamycin-induced albuminuria and kidney injury, indicating that atrasentan is not a viable treatment for adriamycin nephropathy. 

## Materials and Methods

### Animals and treatment protocol

All animal studies were performed in accordance with the recommendations in the Guide for the Care and Use of Laboratory Animals of the NIH. The protocol was approved by the Institutional Animal Care and Use Committee of the University of Pittsburgh Medical Center. Male BALB/c mice (6 to 8-week old) were purchased from Harlan Laboratories (Indianapolis, IN) and housed in the University of Pittsburgh Medical Center animal facility. For the studies examining endothelin-1 and its receptors, mice were subjected to a single intravenous tail-vein injection of adriamycin (10 mg/kg; doxorubicin hydrochloride; Sigma, St. Louis, MO), or saline vehicle for controls. Urine was collected on the day of sacrifice, with mice being euthanized 1, 3, or 5 weeks after adriamycin injection, as indicated. Blood and kidney samples were also obtained at time of sacrifice. 

In the atrasentan experiments we utilized two different protocols. In Protocol 1 (prevention protocol), mice were treated with atrasentan (kindly provided by Abbott Laboratories, Abbott Park, IL) (5 mg/kg, i.p.) daily starting the day prior to adriamycin injection. Seven days after adriamycin injection, mice were sacrificed and samples collected and were compared to a control group that did not receive atrasentan. In Protocol 2 (therapeutic protocol), mice were treated with adriamycin as above, and at seven days after injection, urine was collected and albuminuria assessed. Since adriamycin can cause wide variations in levels of proteinuria, we placed mice into three groups that all had a near-equivalent mean urinary albumin excretion. This protocol ensured that each group was roughly equivalent in terms of glomerular damage and albuminuria, and prevented spurious data that might result from heterogeneity in initial adriamycin-induced injury. The groups were then randomized to one of three treatments arms: 1) vehicle injections; 2) 7 mg/kg atrasentan injections; and 3) 20 mg/kg atrasentan injections. The injections were performed three times weekly for the remainder of the experiment, and were started on day 10. Urine was collected weekly, and sacrifice of mice was performed 35 days after adriamycin administration, and blood and kidney samples were collected. In both Protocol 1 and 2 a corresponding control group that received vehicle injections instead of adriamycin or atrasentan was included for comparison. 

### Drug preparation

Adriamycin was prepared fresh on day of use at a concentration of 1 mg/ml in saline. The drug was dissolved with gentle agitation protected from light, and filtered through a 0.22 micron filter to establish sterility for injection. A 10 mg/kg dose was used for all mice based on effects we have previously described [14]. Atrasentan was initially dissolved in absolute ethanol before dilution in 0.05M NaOH / 0.9% saline. Atrasentan was sterile filtered before use, and delivered as a dose of 5 mg/kg daily in Protocol 1, or as a dose of either 7 mg/kg or 20 mg/kg three times a week in Protocol 2.

### Biochemical measurements

Serum and urine creatinine were measured using a kit from Bioassay Systems (Hayward, CA). A kit from Bethyl Laboratories (Montgomery, TX) was used to determine urinary albumin excretion. Urine albumin was normalized to urine creatinine (mg albumin / mg creatinine).

### Western blots

Protein expression was analyzed by Western blot analysis as described previously [15]. Briefly, total kidney homogenates were prepared in radioimmunoprecipitation assay (RIPA) buffer with protease inhibitor cocktails and protein levels determined by using the BCA protein assay kit (Sigma Aldrich, St. Louis, MO). Equivalent protein amounts were subjected to SDS-PAGE and blotted onto nitrocellulose membranes. Blots were subjected to overnight incubation with primary antibodies followed by secondary antibody incubation for one hour and detected with Supersignal West Pico substrate reagents (Pierce, Rockford, IL). The primary antibodies used were as follows: anti-nephrin (#2OR-NP002, Fitzgerald Industries, Acton, MA), anti-α-smooth muscle actin (α-SMA) (A2547; Sigma, St. Louis, MO), anti-actin (#MAB1501, Chemicon, Billerica MA), and anti-α-tubulin (T9026) (Sigma). 

### Immunofluorescence staining

Frozen tissue sections (5 µm) were prepared from embedded kidneys in optimal cutting temperature (OCT) medium. After blocking for one hour, sections were incubated overnight with anti-nephrin antibody (1:200 dilution, catalog #2OR-NP-002, Fitzgerald Industries, Acton, MA) and anti-WT1 antibody (1:50 dilution, catalog #sc-192, Santa Cruz Biotechnology, Dallas, TX). Sections were then incubated with secondary antibodies conjugated to Cy3 and Cy2, respectively, and imaged with an Olympus Fluoview 500 Confocal Microscope. All images within an experiment were photographed on the same day with identical confocal settings. To determine the number of WT1-positive cells per glomeruli, positively staining cells were counted in 20 glomerular sections from each animal and averaged. 

### Histology and glomerular injury scoring

Paraffin sections (3 µm) were stained with Masson’s trichrome staining reagents from Sigma (St. Louis, MO) according to kit instructions. Blinded gomerular scoring was done by evaluating 20 glomeruli per animal. Scoring was performed with a scale indicating the percentage of the total area of an individual glomerulus that was damaged by adriamycin. The scoring scale was as follows: 0 = no damage; 1 = 1-25% of glomerular area; 2 = 26-50%; 3 = 51-75%; and 4 = 76 to 100% (global damage). 

### Quantitative, real-time reverse transcriptase PCR (qRT-PCR)

RNA was isolated from kidney homogenates using Trizol reagent (Life Technologies, Grand Island, NY). After first strand cDNA synthesis from equivalent starting RNA material for each sample, qRT-PCR was performed using specific primers for ETA, ETB, ET-1, collagen-I, fibronectin, TGF-β1, CTGF, Snail1, and α-SMA using SYBR green reagents and a StepOne PCR instrument from Life Technologies/Applied Biosystems (Grand Island, NY), as described previously [16]. Primer sequences are listed in Table S1. Cycles were run at 95°C for 15 seconds followed by annealing at 60°C for 60 seconds. 

### Statistics

All statistical comparisons were made using a one-way ANOVA. In the event of non-normality of data ANOVA based on ranks was performed. A Dunn’s post-test using pairwise comparison was utilized to determine group differences. *P* < 0.05 was the threshold used for significance.

## Results

### ETA expression is unaffected in adriamycin nephropathy

**Figure 1:**
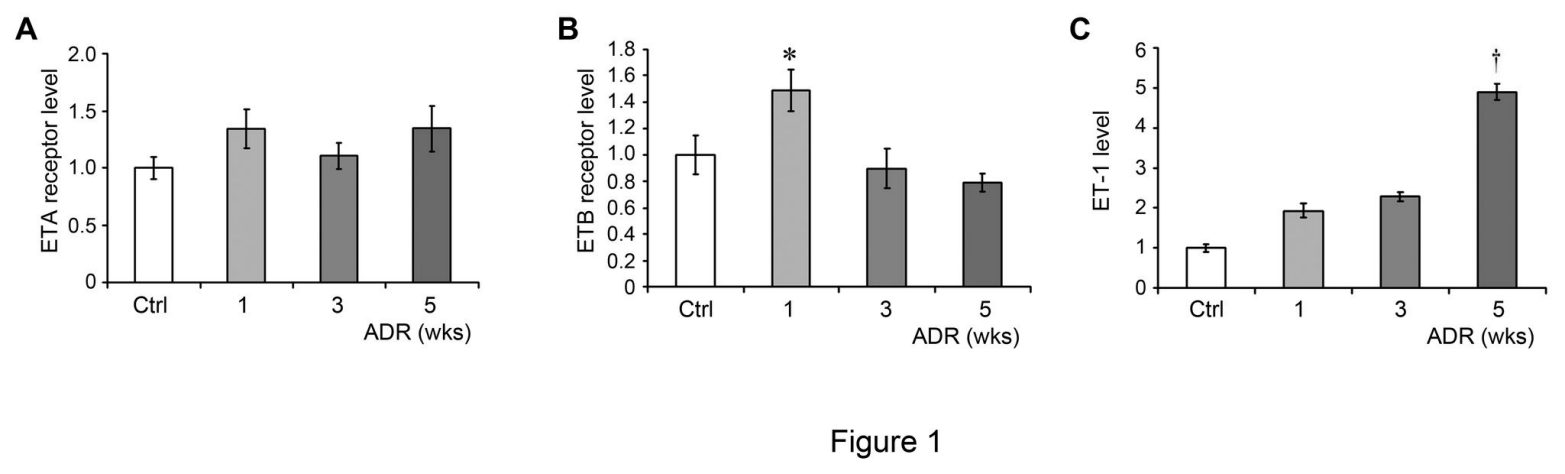
Renal expression of the endothelin system components in adriamycin nephropathy. Mice were treated with saline or adriamycin (10 mg/kg) and sacrificed at the indicated time points. RNA was extracted from harvested kidneys and subjected to quantitative real-time reverse transcriptase polymerase chain reaction (qRT-PCR) analysis. The mRNA expression of the ETA receptor (A), ETB receptor (B), and ET-1 (C) is presented. *P < 0.05 compared to 3 week and 5 week adriamycin groups. †*P* < 0.05 compared to saline controls.

We first examined the effect of adriamycin administration on renal expression of components of the endothelin system. BALB/c mice were treated with 10 mg/kg adriamycin and sacrificed at 1, 3, and 5 weeks. As shown in Figure 1A, ETA receptor gene expression did not change throughout this time course, while ETB had a significant, albeit small, increase in gene expression at 1 week after adriamcyin administration before returning to baseline (Figure 1B). On the other hand, there was a large increase in ET-1 expression after adriamycin administration, but not until 5 weeks after adriamycin injection (Figure 1C). The increase in ET-1 suggested that ETA inhibition could be a viable treatment for adriamycin-induced kidney injury.

### Atrasentan does not prevent proteinuria in adriamycin nephropathy

**Figure 2:**
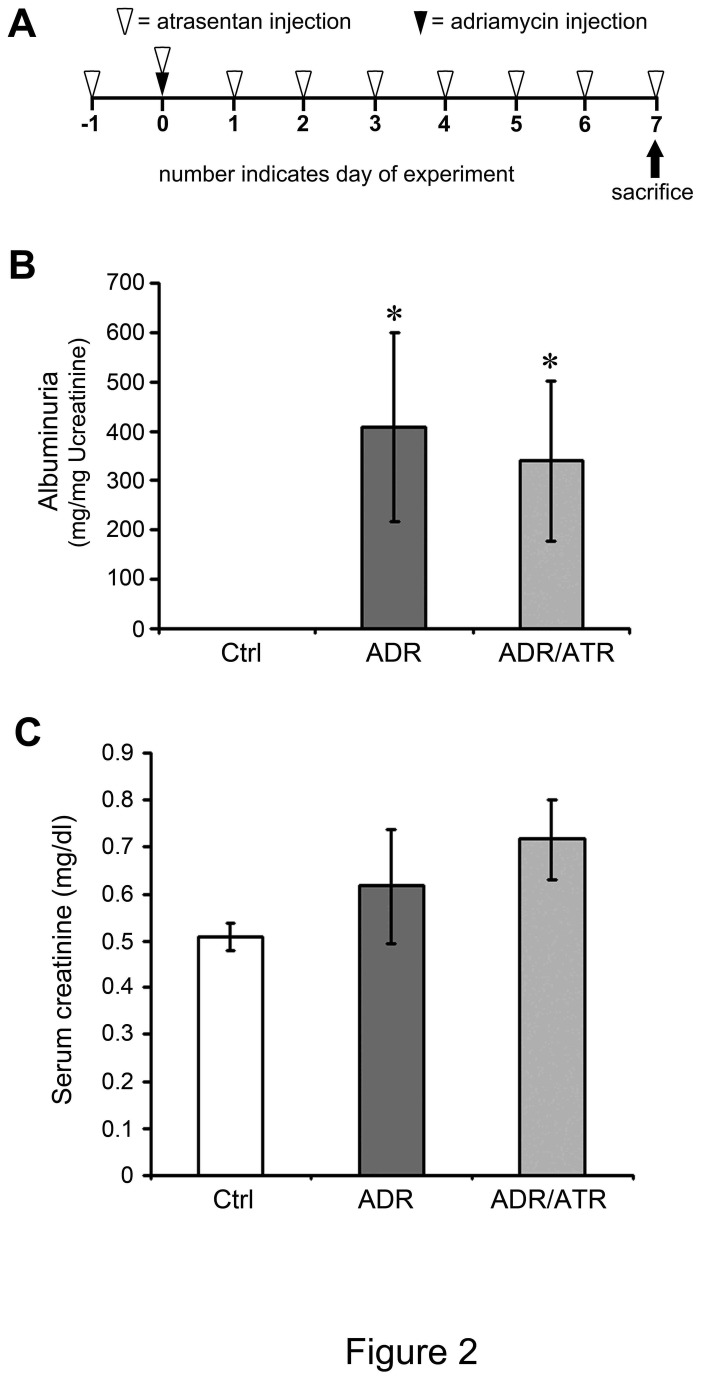
Atrasentan does not prevent proteinuria in adriamycin nephropathy. (A) In this prevention protocol, treatment with atrasentan (5 mg/kg i.p., open triangles) was started one day prior to administration of a single dose of adriamycin (ADR, 10 mg/kg i.v., closed triangle) or saline vehicle (Ctrl). Mice were sacrificed 7 days after adriamycin injection, at which time urine, blood, and tissues were collected. (B) Urinary albumin excretion was determined for each mouse and normalized for urinary creatinine excretion (n=11 for Ctrl group, n=12 for ADR group, and n=12 for ADR / ATR group). *P < 0.05 compared to the Ctrl group. (C) Serum creatinine is shown for all mice in a similar fashion. There was no significant difference between groups at this early time point.

We initially sought to determine the effects of atrasentan in preventing the development of adriamycin nephropathy. By using a prevention protocol (Figure 2A), atrasentan was given to mice starting one day prior to adriamycin administration, at a dose of 5 mg/kg mouse body weight daily. Mice were sacrificed 7 days after adriamycin injection, and urine was collected at the start (day -1) and end of the experiment (day 7). There was no difference in albuminuria at the start of the experiment (control group = 0.201 ± 0.013 mg/mg creatinine; adriamycin group = 0.889 ± 0.673; adriamycin/atrasentan group = 0.269 ± 0.656). In agreement with previous data, albuminuria was increased after adriamycin treatment compared to normal controls (Figure 2B) [14]. However, there was no significant difference between adriamycin-treated mice and adriamycin/atrasentan-treated mice (n=12 for each group). Serum creatinine also did not differ after adriamcyin with or without atrasentan (Figure 2C). 

### Atrasentan is unable to prevent podocyte dysfunction

**Figure 3:**
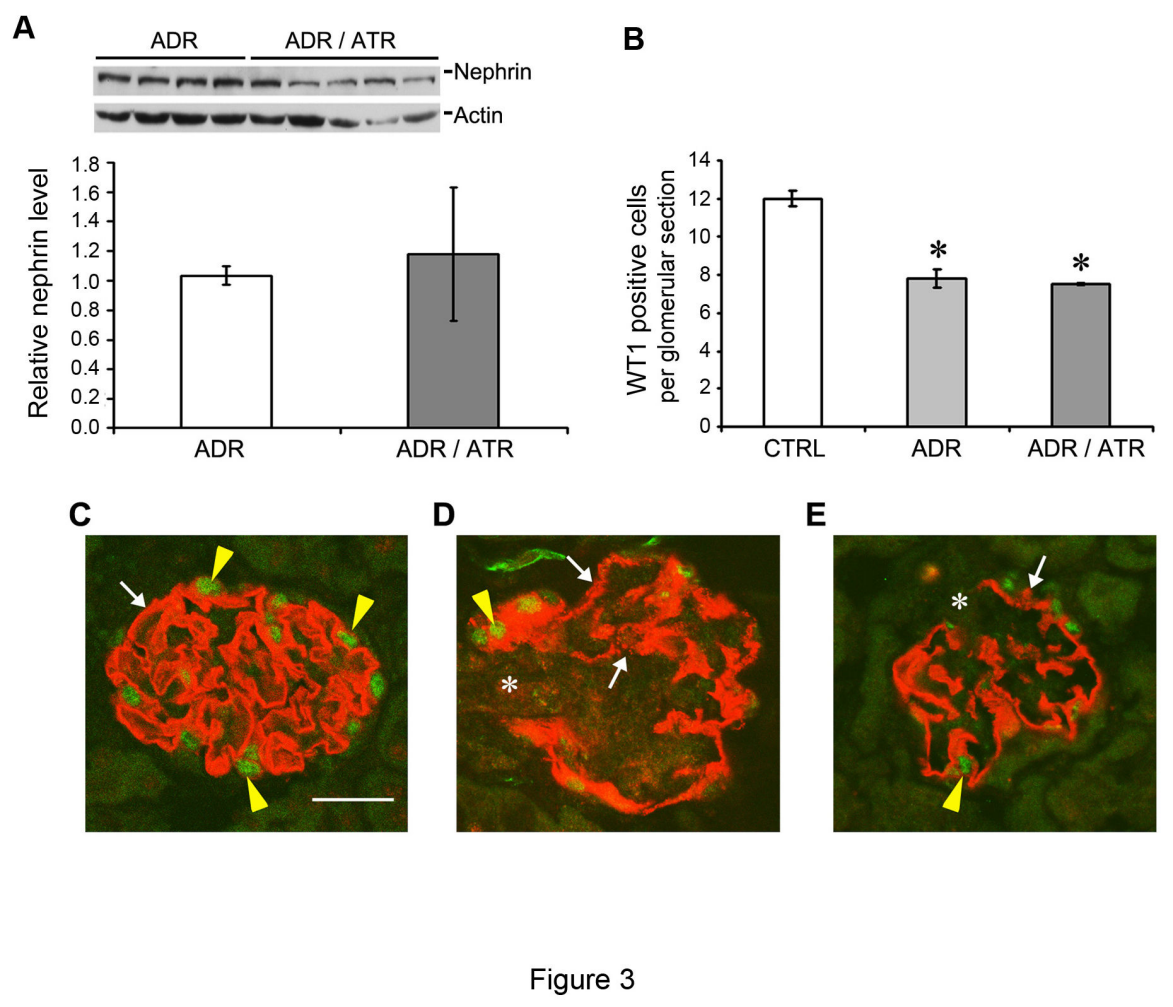
Podocyte injury is not attenuated by atrasentan in the prevention protocol. Kidneys from the mice treated in Figure 2 were assessed for podocyte markers. (A) Western blot showing no overall change in nephrin levels from whole kidney homogenates. Densitometry was normalized to actin as a loading control. (B) WT1 positive nuclei were counted in 20 glomerular sections for each mouse (n=4) after immunofluorescence staining. *P < 0.05 compared to control. (C) Normal glomerular immunofluorescence staining for nephrin (red) and WT1 (green). Note the linear nephrin staining (white arrow) and abundant WT1-positive nuclei (yellow arrowheads). Scale bar equals 20 μm. (D) Representative micrograph showing the glomerulus from a mouse treated with adriamycin (without atrasentan) and sacrificed at 7 days. Note that the nephrin staining is not uniformly linear and appears speckled in numerous areas (white arrows) and is completely disrupted in other areas (asterisk). There is also a decrease in WT1-positive nuclei (yellow arrowhead). (E) Glomerulus from an adriamycin-treated mouse that has been cotreated with atrasentan as per the protocol in Figure 2A, showing similar losses of linear nephrin staining (white arrow) as well as complete disruption of nephrin (asterisk) and similar reductions in WT-1 nuclei (yellow arrow).

Since podocyte dysfunction is a key feature of adriamcyin nephropathy, we next examined whether there is an effect of atrasentan on podocytes in our model. To do this, we examined nephrin and WT1 (Wilms tumor 1) levels. Nephrin protein levels did not change significantly when atrasentan was added to adriamycin treatment in mice when examined by western blot analyses (Figure 3A). Similarly, when counting WT1 positive cells per glomerular section, we detected a decrease in WT1 expression after adriamycin, but no significant difference when atrasentan was added to adriamycin (Figure 3B). Immunofluorescence staining also showed that control mice had a linear pattern of nephrin staining along the slit diaphragm, which indicates normal nephrin distribution and podocyte function. However, the adriamycin-treated mice had non-linear granular staining which was not improved with the addition of atrasentan. This non-linear staining indicates aberrant nephrin distribution and podocyte dysfunction. Similarly, while control mice had abundant WT-1 nuclear staining, this staining was decreased in adriamycin mice and was not significantly different in the adriamycin / atrasentan mice (Figure 3, C-E). These results indicated that atrasentan was incapable of preventing initial podocyte injury when exposed to adriamycin in vivo. 

### Atrasentan fails to ameliorate established proteinuria and podocyte injury

**Figure 4:**
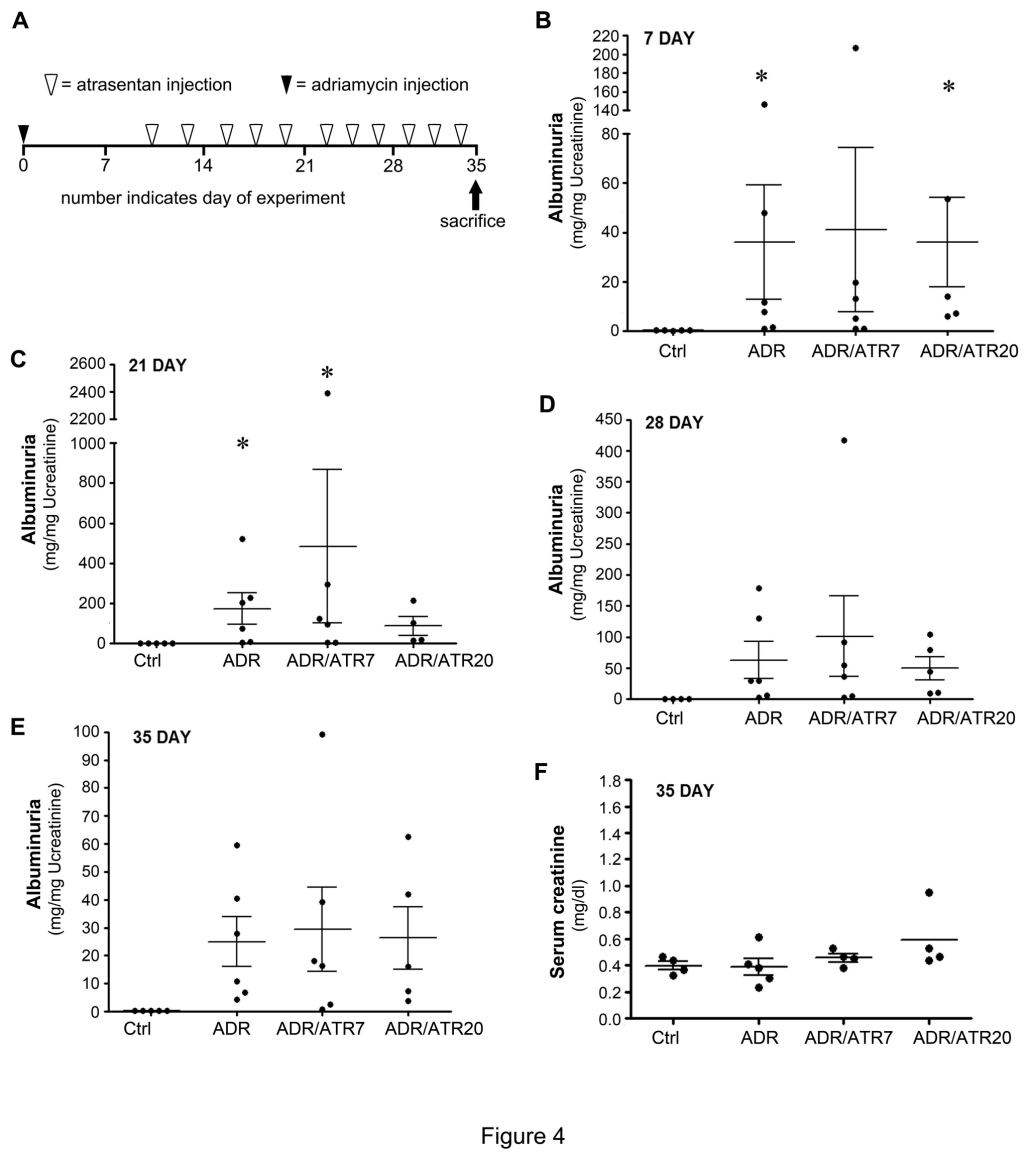
Atrasentan does not ameliorate established proteinuria. (A) In this therapeutic protocol, mice were first treated with adriamycin on day 0 (10 mg/kg, closed triangle). After evaluation of albuminuria on day 7, mice were randomized into groups as described in Materials and Methods to receive vehicle (ADR, n=6) or atrasentan at either 7 mg/kg (ADR/ATR 7, n=6) or 20 mg/kg (ADR/ATR 20, n=5) which are designated as open triangles. Atrasentan was started on day 10 and given three times weekly until sacrifice at 35 days. Control mice (Ctrl) that did not receive any adriamycin or atrasentan were also included. (B) Albuminuria at 7 days post-adriamycin. Each dot represents an individual mouse. Note that the mice are grouped such that similar levels of albumin excretion were included in each arm of the study. Albuminuria was then measured at day 21 (C), day 28 (D), and day 35 (E). (F) Serum creatinine was also assessed at 35 days. *P < 0.05 compared to control group only.

**Figure 5:**
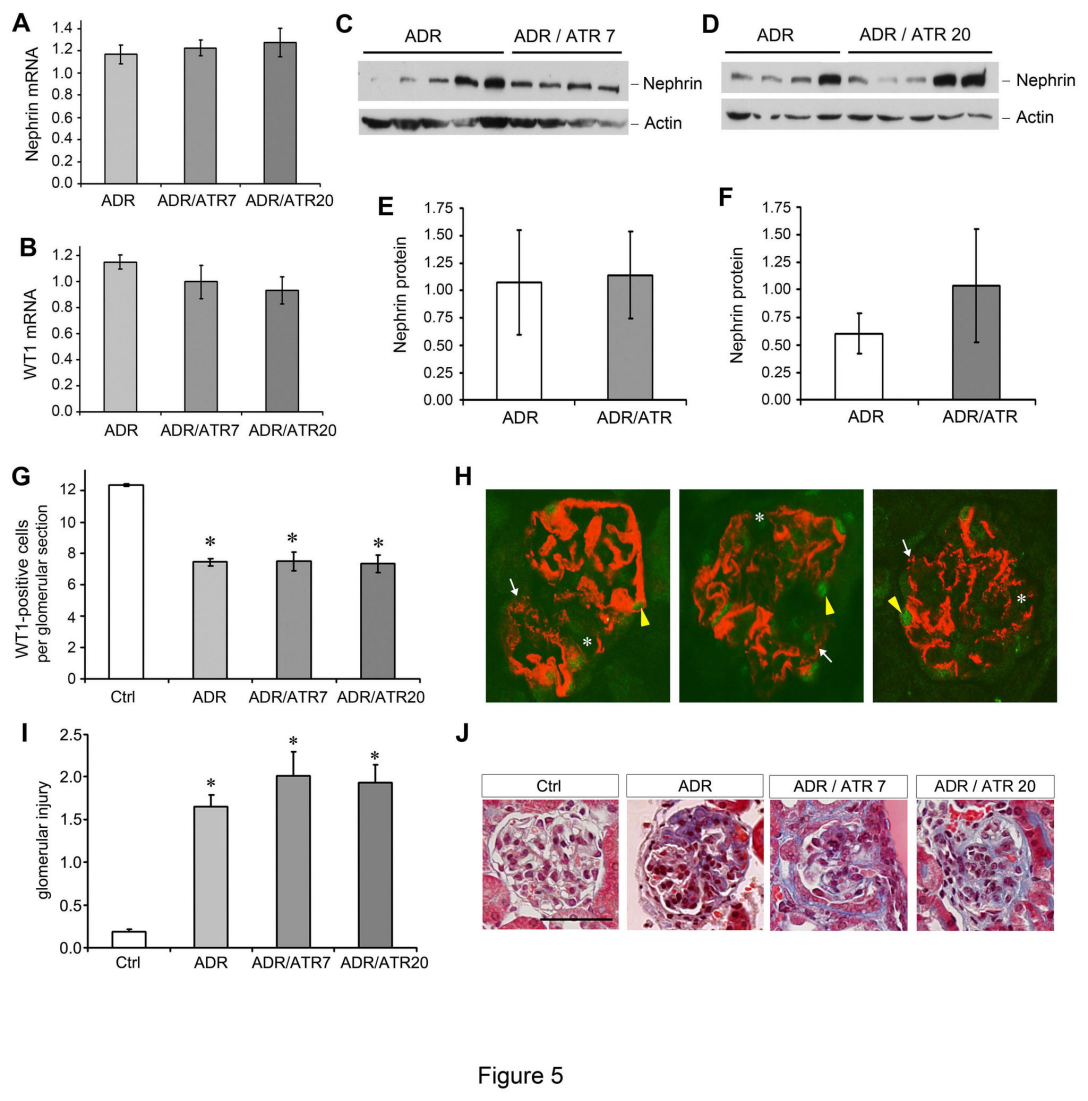
Atrasentan does not ameliorate established podocyte injury in adriamycin nephropathy. Mice were treated as in Figure 4A. (A, B) qRT-PCR analysis showed nephrin (A) and WT1 (B) mRNA expression. ADR, adriamycin alone; ADR/ATR7, adriamycin-treated mice that also received atrasentan at 7 mg/kg; ADR/ATR20, adriamycin-treated mice that also received atrasentan at 20 mg/kg. (C-F) Western blots and densitometry for nephrin expression in different groups as indicated. (C and E) Atrasentan at 7 mg/kg; (D and F) Atrasentan at 20 mg/kg. (G) WT1-positive nuclei in 20 glomerular sections from each animal (n=3 for each group) were counted as in Figure 3. *P < 0.05 compared to control group only. (H) Representative glomerulus from mouse treated with adriamycin and sacrificed at 35 days (left panel). Atrasentan at a dose of 7 mg/kg (middle panel) or 20 mg/kg (right panel) did not improve the loss of linear staining for nephrin (white arrows), the areas of complete nephrin disruption (asterisk), or the decrease in WT1-positive nuclei (yellow arrowheads). (I) Histologic scoring of 20 glomeruli per animal (n=4 for Ctrl group and n=5 for all other groups) reveals no difference when atrasentan is added to adriamycin treatment. * *P* < 0.05 compared to control group only. (J) Representative images of glomeruli from Ctrl and treatment groups, as indicated. Note a lack of improvement in glomerular histology with atrasentan treatment. Scale bar equal 50 μm.

Given that ET-1 is induced at a late time point in this model (Figure 1), we next tested whether blockade of ETA by atrasentan at late time points could ameliorate proteinuria and slow the progression to CKD. Therefore, we designed a therapeutic protocol (Figure 4A). Mice were subjected to treatment with saline or adriamycin, with urine collected at 7 days. Mice were then randomized into groups such that mean albuminuria was similar between each group at 7 days after adriamycin injection (Figure 4B). This randomization based on albuminuria level was used because of a wide inter-animal variability in this model [13]. We utilized two different doses of atrasentan, given as 7 mg/kg or 20 mg/kg three times weekly starting on day 10 after adriamycin. As shown in Figure 4, C through E, there was no improvement of proteinuria with either dose of atrasentan when compared to vehicle controls at 21, 28, or 35 days after adriamycin injection. These conclusions were unchanged even if we eliminated the few mice exhibiting markedly higher albuminuria than their counterparts (data not shown). Similarly, serum creatinine was unaffected by atrasentan, although at this time point the creatinine did not increase after adriamycin (Figure 4F). When podocyte-specific markers were assessed, there were no differences in overall nephrin expression in atrasentan-treated mice (at either dose) compared to vehicle controls (Figure 5, A-D). In terms of WT1-positive cells there was again no significant difference in adriamycin-treated mice given either dose of atrasentan (Figure 5G). This remained true even if the WT-1 positive cells were normalized to the total number of nuclei per glomerular section (data not shown). The immunofluorescence staining for nephrin and WT1 was not improved by atrasentan (Figure 5H). Finally, when we assessed glomerular damage histologically, we also did not find any overall improvement when comparing adriamcyin-treated mice with mice that also received atrasentan (Figure 5, I-J). Collectively, we were unable to detect any improvement in glomerular damage parameters with atrasentan. 

### Atrasentan does not improve fibrotic lesions in adriamycin nephropathy

**Figure 6:**
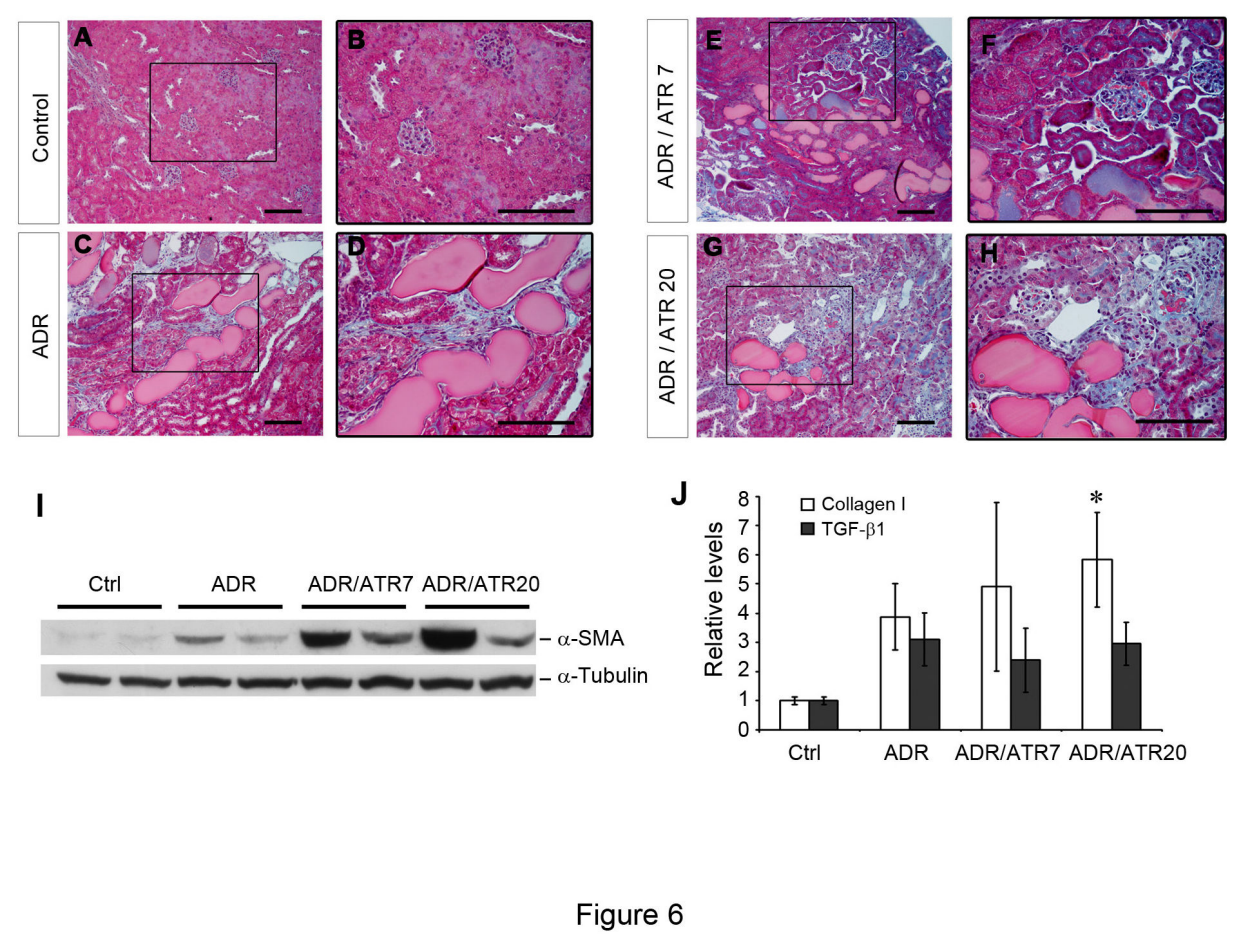
Atrasentan does not affect morphologic injury and fibrogenic gene expression in adriamycin nephropathy. Masson’s trichrome staining was performed to determine levels of histologic injury in the mice at 35 days after adriamycin injection. A saline-treated control that received neither adriamycin nor atrasentan is shown in Panel A with enlargement of the boxed area in Panel B. Scale bar equals 100 μm in each figure. Histology from a mouse treated with adriamycin is shown in Panel C with corresponding enlargement in D. Adriamycin with atrasentan at 7 mg/kg is shown in Panel E with enlargement in F. Adriamycin with atrasentan at 20 mg/kg is shown in Panel G with enlargement in H. In Panel I, Western blot for α-smooth muscle actin (α-SMA) is shown for different groups as indicated with α-tubulin as a loading control. In Panel J, qRT-PCR was performed for assessing mRNA expression of collagen-I and TGF-β1. * *P* < 0.05 for ADR / ATR20 compared to saline Ctrl.

Although glomerular damage and albuminuria are the primary outcomes of our study, we elected to examine indicators of chronic renal injury as a secondary endpoint. At 35 days, histology was examined with Masson’s trichrome staining, which stains collagen deposition with blue color. There was an increase in damaged tubules and collagen deposition in adriamycin-treated mice compared to normal controls (Figure 6, A versus C). Although there was some variability between animals, there was no overall difference in histological damage between vehicle and atrasentan groups (Figure 6, G-H). In agreement with these results, the expression of α-smooth muscle actin (α-SMA), a marker of myofibroblast activation, was not reduced by atrasentan treatment (Figure 6I). 

Utilizing quantitative RT-PCR (qRT-PCR), we further examined the expression of numerous genes such as collagen-I, TGF-β1, fibronectin, α-SMA, connective tissue growth factor (CTGF), and Snail1, which are all implicated in renal fibrosis through extracellular matrix accumulation, epithelial-mesenchymal transition, or fibroblast activation [17]. As shown in Figure 6J, atrasentan did not affect renal mRNA expression of collagen-I and TGF-β1 at 5 weeks after adriamycin injection. Similarly, atrasentan also did not inhibit the mRNA expression of other fibrosis-related genes including fibronectin, α-SMA, CTGF and Snail1 (data not shown). 

## Discussion

Atrasentan has been shown to reduce albuminuria in a variety of experimental kidney injuries, presumably due to blockade of ET-1 signaling through the pathologic ETA receptor. Atrasentan has been shown to reduce urinary protein excretion in rodent experimental models of both hypertension and diabetes [5,18,19]. These experimental findings are accompanied by human studies which also revealed reduced albuminuria in diabetic patients receiving atrasentan [10].

Based on these observations, we originally hypothesized that atrasentan would prevent the albuminuria associated with adriamycin nephropathy, a model for human FSGS. To our surprise, we were unable to detect any improvement in albumin-to-creatinine ratios in this model when atrasentan was delivered daily prior to the start of injury in a 7-day acute prevention model. Similarly, there was no improvement in albuminuria when atrasentan was given after the onset of injury in a chronic 35-day protocol. Consistent with these findings, there was no effect on histologic glomerular damage and overall nephrin and WT1 expression in glomerular podocytes by atrasentan in vivo. In our chronic therapeutic protocol, we were also unable to detect any improvement in serum creatinine, histologic damage, or expression of fibrosis-related genes. 

The reasons for the lack of efficacy of atrasentan in our adriamycin model may be complex. First, we were unable to detect a difference in ETA receptor expression at 1, 3, or 5 weeks after adriamycin injection (Figure 1A), suggesting that ETA activation is not a primary mechanism leading to proteinuria in this model. Furthermore, there was no change in ET-1 expression until very late in the course of disease, 5 weeks after adriamycin injection (Figure 1C). The lack of an increase in either ET-1 or ETA receptor prior to the 5 week timepoint could explain why an ETA-specific receptor inhibitor delivered during this time was ineffective in preventing albuminuria. However, we cannot completely exclude the possibility that atrasentan might be effective in preventing albuminuria after 5 weeks in this model. 

Second, in terms of glomerular hemodynamics, ETA receptor activation is purported to lead to altered afferent and efferent arteriolar vasoconstriction [1,20]. These effects would affect intraglomerular pressures and could lead to glomerular hyperfiltration, which in turn could alter urinary albumin excretion and cause glomerular injury. Glomerular hyperfiltration does occur in subtotal renal ablation and diabetic nephropathy [21,22], and atrasentan is indeed capable of ameliorating injury in these models [8,9]. However, it is unclear if there is really increased intraglomerular pressures in adriamycin nephropathy, as earlier studies utilizing intricate single nephron micropuncture techniques have shown that adriamycin does not lead to significant hyperfiltration in rat glomeruli *in vivo* [23]. As such, there would be no benefit for atrasentan in improving intraglomerular pressures. 

Other pathologic mechanisms for ET-1 must also be considered. It has been proposed that podocyte cytoskeletal disruption and endothelin activity are related events [24]. Our study was not targeted at identifying such cytoskeletal rearrangements, which would require specialized imaging techniques. However, nephrin is a common marker of podocyte injury and slit-diaphragm integrity. We were able to measure this key protein and could not find any differences by several techniques (Western blotting, qRT-PCR and immunostaining). In addition, we demonstrated that levels of WT1, which is normally expressed by podocytes but is downregulated in injury, could not be sustained with the use of atrasentan, suggesting that atrasentan was not able to ameliorate podocyte dysfunction in our model. ET-1 is also thought to play a role in the profibrotic response, particularly in upregulation of TGF-β and other fibrosis-related genes [25]. We attempted to assess expression of these genes, but did not find a significant difference between the vehicle and atrasentan groups at 35 days after adriamycin injection. 

A potential concern is the dosing regimen we utilized for atrasentan. Numerous studies utilized a dose of 5 mg/kg/day in diabetic nephropathy [5], hypertensive glomerular injury [18], congestive heart failure [26], and hypertension [27]. This is the same dose utilized in our acute prevention protocol. For our chronic studies we utilized either of two doses of atrasentan (7 mg/kg or 20 mg/kg) given three times weekly (cumulative weekly doses of 21 mg/kg or 60 mg/kg, respectively), which provided similar doses to a chronic protocol from a previous report in which mice were given 5 mg/kg/day for 5 days each week (cumulative dose of 25 mg/kg per week) [27]. While the cumulative weekly doses are similar or even higher in our study, we must acknowledge that the thrice weekly atrasentan dose may have different results compared to a lower dose given 5 times per week in these experiments. It should be noted that our initial 7-day experiment did include a daily dosing regimen with an adequate dose of atrsesntan that was initiated before the onset of adriamycin injury. The result from the shorter experiment in the prevention protocol was still negative, suggesting lack of effect of atrasentan in this model.

Of note, our use of intraperitoneal (i.p.) injections is another difference from earlier studies which predominantly utilized oral atrasentan. Previous studies with atrasentan in rats revealed approximately 35% bioavailability of oral atrasentan with biological effects persisting to at least 24 hours [4]. While we could not find precise pharmacokinetic data for i.p. doses, it was reasonable to assume blood concentrations and duration of effect of the drug at least as high as the oral route. Further, i.p. dosing of atrasentan has been utilized by other investigators [28]. Nonetheless, we must acknowledge this as a difference between our paper and some prior studies that may have affected our results. 

In our chronic 35-day experiment, we elected to start atrasentan injections after the initiation of adriamycin injury for two reasons. First, this is similar to the clinical situation in which patients with established renal injury receive a medication to ameliorate disease. Second, ET-1 induction is a late event in this model (Figure 1). In addition, this therapeutic protocol allowed us to randomize mice into homogenous groups based on their urinary albumin excretion at 7 days after adriamycin injection, as proteinuria and renal damage display a wide inter-animal variability in this model [13]. We believed that this was a way to reduce statistical heterogeneity caused by variations in albuminuria, akin to the use of inclusion criteria in clinical trials. In spite of this, we were still unable to find any significant albuminuria differences between groups. 

In conclusion, atrasentan in our experimental protocol was incapable of ameliorating albuminuria and podocyte injury associated with adriamycin nephropathy, a model of human FSGS. The causes for this may be related to the characteristic features of this particular model of proteinuric kidney disease, in which ETA expression is unaltered. Our results suggest that blockade of ETA signaling by atrasentan may not be effective in a subset of chronic kidney diseases wherein activation of the endothelin system is not a primary cause of proteinuria and kidney damage. 

## Supporting Information

Table S1 **Primer sequences used in this study.**
(DOC)
