# Supplementary material for: Endothelin Receptor A Blockade Is an Ineffective Treatment for Adriamycin Nephropathy
Source: PLoS One. 2013 Nov 12;8(11):e79963. doi: 10.1371/journal.pone.0079963 (PMC3825716; doi:10.1371/journal.pone.0079963)
Supplement: Table S1 — Primer sequences used in this study. (DOC) [file pone.0079963.s001.doc]

**Table S1. Primer sequences** used in this study

|  | Primer 1 | Primer 2 |
| --- | --- | --- |
| Endothelin-1 | GGCCCAAAGTACCATGCAGA | GATGGCCTCCAACCTTCGTA |
| ETA | GAAGGACTGGTGGCTCTTTG | CTTCTCGACGCTGTTTGAGG |
| ETB | CGCTCTGTATTTGGTGAGCA | AGTGAGATTCGGCGAGTGTT |
| Collagen-1 | ATCTCCTGGTGCTGATGGAC | ACCTTGTTTGCCAGGTTCAC |
| Fibronectin | CGAGGTGACAGAGACCACAA | CTGGAGTCAAGCCAGACACA |
| α-SMA | GAGGCACCACTGAACCCTAA | CATCTCCAGAGTCCAGCACA |
| TGF-β | GTGGAAATCAACGGGATCAG | GTTGGTATCCAGGGCTCTCC |
| CTGF | CCTGGTCCAGACCACAGAGT | TTTTCCTCCAGGTCAGCTTC |
| Snail1 | ATTCTCCTGCTCCCACTGC | GACTCTTGGTGCTTGTGGAG |
